# Exploring the Relationship Between Conspiracy Theory Beliefs and Adherence to Government Guidelines During the COVID-19 Pandemic: The Role of Perceived Control and Trust in Social Media and Traditional Sources of Information

**DOI:** 10.3390/healthcare13222915

**Published:** 2025-11-14

**Authors:** Maria Stella Epifanio, Vittoria Spicuzza, Martina Riolo, Emanuele Cusumano, Marco Andrea Piombo, Sabina La Grutta

**Affiliations:** Department of Psychology, Educational Science and Human Movement, University of Palermo, 90133 Palermo, Italy; mariastella.epifanio@unipa.it (M.S.E.); vittoria.spicuzza@unipa.it (V.S.); martina.riolo@unipa.it (M.R.); emanuele.cusumano97@gmail.com (E.C.); sabina.lagrutta@unipa.it (S.L.G.)

**Keywords:** COVID-19, conspiracy beliefs, misinformation, trust in information sources, social media, adherence to public health guidelines, perceived control, psychological well-being

## Abstract

**Background:** The COVID-19 pandemic, declared to be over by the World Health Organization (WHO) on 5 May 2023, significantly impacted global physical, mental, economic, social, and political conditions. Since the onset of the pandemic, conspiracy theories have surged globally, facilitated by the Internet and social media. Conspiracy thinking is associated with mistrust in traditional sources of information, such as newspapers and news/TV programs, and lower adherence to public health guidance. However, there is limited understanding of how these beliefs are reflected in specific health-related behaviors and the mediating variables involved. **Objective:** The study aims to analyze the relationship between the belief in conspiracy theories, perceived personal control, and psychological well-being during the COVID-19 pandemic in Italy to understand how belief in conspiracy theories may contribute to less adherence to government guidelines and the role of factors such as personal control, trust in social media, and traditional sources of information in this relationship. **Methods:** In total, 437 Italian adults (296 women, 140 men, 1 non-binary; M_age = 31.41, SD = 13.32) completed measures of well-being, perceived control, use/trust of traditional vs. social-media sources, conspiracy beliefs, and adherence. **Results:** Well-being correlated positively with perceived control and social-media trust. Perceived control correlated positively with social-media trust and negatively with traditional-source trust. Adherence correlated positively with traditional-source trust and negatively with all conspiracy measures. Mediation showed an indirect effect of conspiracy beliefs on lower adherence only via reduced trust in traditional sources. In contrast, no indirect effects were found via social-media trust or perceived control. **Conclusions:** Conspiracy beliefs undermine adherence primarily by eroding trust in traditional information. Risk communication should rebuild institutional trust and tailor messaging across both social and traditional channels, taking into account psychological factors.

## 1. Introduction

On 5 May 2023, the World Health Organization (WHO) declared the end of the COVID-19 pandemic, marking more than three years since its inception. In Italy, on 7 August 2023, the obligation of isolation for COVID-19-positive people was abolished. This global event has inevitably resulted in a profound change in the lives of billions of individuals, and the effects on people’s physical and mental health, as well as their economic, social, and political conditions, are well known [1].

For this reason, it is possible to count the COVID-19 pandemic among the events that constitute a social crisis, as defined by van Prooijen and Douglas: “a rapid and impactful social change that challenges existing power structures, norms of conduct, or even the existence of specific people or groups [2].” Since the start of the COVID-19 pandemic, conspiracy theories have proliferated worldwide on the Internet and social media [3].

The widespread use of fake news, such as conspiracy theories, through non-traditional information sources, has led the Director-General of the WHO to state that “We’re not just fighting an epidemic; we’re fighting an infodemic. Fake news spreads faster and more easily than this virus, and is just as dangerous” [4]. Previous findings [5,6] show that conspiracy thinking is associated with mistrust in traditional media and lower compliance with public health guidance, for instance: maintaining social distance, handwashing, and vaccines. At the same time, conspiracy theories increase risky behaviors, such as the case of the consumption of highly concentrated alcohol to kill the coronavirus, which resulted in various deaths and hospitalizations worldwide [7,8].

The COVID-19 pandemic has been particularly characterized by uncertainty about the origin of SARS-CoV-2, the severity of the disease, and the lack of specific treatments. For example, the COVID-19 vaccine was not well-received by the population. Low vaccine acceptance was associated with low levels of education and awareness, as well as inefficient government efforts and initiatives [9]. A few systematic reviews analyzing worldwide vaccine hesitancy and acceptance rates have also recently surfaced [9]. The vaccine acceptance rate in Europe ranged from 27% to 91.5%. Neumann-Bohme et al. [10] studied seven European countries, including Denmark, the UK, Portugal, the Netherlands, Germany, France, and Italy. Vaccine hesitancy was related to mistrust in a vaccine that had been prepared in a very short amount of time. Another study surveyed parents about vaccinating their children and found that uncertainty about vaccine safety was the primary factor contributing to vaccine rejection [11]. Moreover, prior to the pandemic, another study involving 67 countries found that Italy was among the countries with the highest levels of skepticism and doubt regarding the effectiveness of vaccinations [12].

In these extraordinary circumstances, it has been highlighted that a possible relationship exists between negative information on COVID-19 vaccines seen on social media and low acceptance, suggesting a relevant role played by misinformation [13]. Indeed, the pandemic has been accompanied by an unprecedented infodemic, i.e., an overabundance of information that makes it hard for people to find trustworthy sources and reliable guidance when they need it. According to van Prooijen and Douglas [14], conspiracy theories find fertile ground to develop in such a context.

Conspiracy theories provide, indeed, alternative explanations for impactful crisis events in the world, such as pandemics, wars, or natural disasters [14], and play an important defensive role against uncertainty and lack of control over events that result from living in particular historical moments: conspiracy theories would therefore respond to the pressing need for answers, allowing people to form judgments about events that happen [14]. Moreover, conspiracy theories could contribute to satisfying social and psychological needs, such as the epistemic need to understand the environment, feel safe, and maintain a positive image of oneself and one’s group. More specifically, conspiracy theory beliefs could increase when one’s image is threatened and there is a strong need to feel unique [15,16]. The condition of missing control, or being faced with unpredictable threats, leads people to see illusory patterns in random events as a way of introducing order and predictability to life [15]. The high need for control has been related to distortions of objective reality because this kind of illusory pattern acts as a compensatory mechanism to restore feelings of control and, consequently, well-being [17].

On the other hand, despite conspiracy beliefs being a response to anxiety and uncertainty, individuals’ belief in conspiracy theories has been related to mental disorders, lower psychological well-being, and maladaptive behavior [18,19]. According to van Prooijen [14], a plausible explanation is that the epistemic assumption that a small group of powerful people conspires in the shadows against common citizens reduces feelings of lack of control and anxiety and sometimes can even increase them, creating a sort of paradox. Moreover, despite the fact that conspiracist sub-communities (easy to find on social media) respond to a social need of belonging [15], individuals who believe in conspiracy theories are also vulnerable to stigmatization and social rejection, which predict significant social consequences such as job loss (think of health-care workers who refused the vaccine and were disbarred from their professional association) [14]. In summary, these theories may reduce feelings of lack of perceived control in one way (e.g., “SARS-CoV-2 is only the flu”) but reinforce these feelings in another way (e.g., “the belief that the government wants to establish a health dictatorship”). In other words, conspiracy beliefs and negative feelings mutually reinforce each other in a sort of loop of anxiety and mistrust [15].

In the specific context of Italy, during the COVID-19 pandemic, the most popular conspiracy theories were as follows: the belief that SARS-CoV-2 was created in the laboratory, the belief that COVID-19 is only a flu, and the belief that COVID-19 was introduced to limit citizens’ freedom and establish a health dictatorship. Also, regarding vaccines, we took into account the most widespread conspiracy theories at the time of the data collection: the belief that the vaccine was introduced only to make Pharma companies richer, the belief that physicians cannot tell the truth to avoid the possibility of losing their jobs, and the belief that the vaccine is more harmful than COVID-19 itself.

The literature suggests that belief in conspiracy theories is often related to socio-demographic variables such as level of education [20], age [21], maladaptive personality traits [22], lack of control, and lower well-being [23]. In addition, according to the study by Nazlı, Ş. B. et al. [24], those who believe in conspiracy theories have a greater hesitation towards the vaccine.

Even since 2019, there has been a rapid growth in research about the possible negative effects of belief in conspiracy theory: anti-science attitudes, political violence, and general mistrust of established institutions [15]. However, few studies have focused on identifying the mechanisms that lead individuals who believe in conspiracy theories to engage in risky behaviors and not follow government guidelines. Existing literature largely concentrates on the factors that make people believe in conspiracy theories but lacks a thorough examination of how these beliefs translate into specific health-related behaviors and the role of mediating variables in this process.

For these reasons, in this study, starting from the perspective of social and clinical psychology, we are interested in understanding how belief in conspiracy theories, as a response to strong feelings of uncertainty and lack of control, may contribute to trust of traditional sources and social of information and, consequently, to increased risk behaviours in terms of low adherence to public health guidance that have hindered measures to contain the spread of the COVID-19.

Therefore, the present study aims to analyze the relationship between belief in conspiracy theories related to COVID-19 and vaccines, perceived personal control, perceived individual psychological well-being status, adherence to government guidance, perceived threat against the COVID-19 vaccine, and, finally, the use of main sources of information, as well as the trust placed in them.

The hypotheses that guide this study are as follows: first, belief in conspiracy theories is more prevalent in those who exhibit lower perceptions of personal control and lower well-being; second, those who believe in conspiracy theories showed lower adherence to health behaviors; third, conspiracy theories are most prevalent in those individuals who most use and trust “non-traditional” but web-related media, such as social media [25,26], and finally, perceived control and trust in traditional and social media sources have a mediating role in the relationship between belief in conspiracy theories and health-related behaviors during pandemic. Accordingly, we hypothesized an indirect pathway between conspiracy beliefs and lower adherence, primarily through reduced trust in traditional information sources and, secondarily, through lower perceived control, and trust in social media, tested via a serial multiple-mediator model.

## 2. Materials and Methods

### 2.1. Participants and Procedures

An online cross-sectional survey was conducted using the Survey Platform (Google Forms; Google LLC, Mountain View, CA, USA). The survey began on 6 December 2021, and lasted approximately four weeks. The sample was recruited via a snowball sampling strategy. The link to the survey was spread through various channels, such as email and social networks, such as Facebook and WhatsApp (Meta Platforms, Inc., Menlo Park, CA, USA), to reach a broader audience. Upon accessing the survey link, participants were presented with a brief introduction that outlined the aims of the study. This introduction also included information about the study’s purpose and the procedures involved. Participants were required to provide electronic informed consent before proceeding with the survey. The consent form assured participants of maximum confidentiality in the handling and analysis of their responses. The survey took approximately 10 min to complete. Participation was voluntary and free of charge. To guarantee anonymity, no personal data, which could allow the identification of participants, was collected. The study was conducted in accordance with the Declaration of Helsinki and approved by the Ethics Committee of the Institution of the first author (25 May 2020, n.4/2020).

The final sample consisted of 437 Italian adults (296 females, 140 males, and one no-binary; Mean age = 31.41, SD = 13.32). An a priori power analysis was conducted in G*Power 3.1 (Heinrich Heine University Düsseldorf, Düsseldorf, Germany) to guide recruitment for the core analyses that motivated the design. For Pearson correlations among the study variables (two-tailed, α = 0.05, 1 − β = 0.80), we targeted a small-to-moderate association (*r* = 0.20), which yields a required *N* ≈ 194. For independent-samples comparisons between believers and non-believers on key outcomes (two-tailed *t* tests, α = 0.05, 1 − β = 0.80), we targeted a small-to-moderate effect (Cohen’s *d* = 0.30). We considered potential group imbalance which places the required total sample in the low–mid 300 s. Our previously reported minimum of *N* = 129 reflected an initial planning scenario assuming medium effects (e.g., *r* = 0.25) under more favorable balance; nevertheless, we recruited *N* = 437 to ensure adequate power across the full set of planned tests. People were included in the study if they were Italian, living in Italy during the Pandemic, and at least 18 years old.

### 2.2. Measures

#### 2.2.1. Demographic and COVID-Related Information

An ad hoc questionnaire was created to collect demographic data (such as sex, age, marital status, education level, occupational status, and region of residence in Italy) and COVID-related information (i.e., having tested positive for COVID-19 or knowing someone who tested positive for COVID-19). Moreover, in this section, participants were asked if they had undergone the vaccine.

#### 2.2.2. Psychological General Well-Being

The Psychological General Well-Being Index (PGWBI) in its Italian validated version [27] is a measure of the level of subjective psychological well-being. In detail, it assesses self-representations of intrapersonal affective or emotional states reflecting a sense of subjective well-being or distress and thus captures what we could call a subjective perception of well-being. The PGWBI consists of 22 self-administered items, rated on a 6-point Likert scale, which assesses the psychological and general well-being of respondents in six domains: anxiety, depression, positive well-being, self-control, general health, and vitality. For the purpose of this study, only the global Well-Being score was used, and it was computed by the sum of all item scores (higher scores represent higher global Well-Being). In this study, only the general well-being score was used, and this scale showed excellent internal reliability (Cronbach’s alpha = 0.91).

#### 2.2.3. Perception of the Control—Loss of Control

According to the theorizations concerning the compensatory control mechanism construct, the perception of the personal control of the study participants was measured during the pandemic; to do this, a 10-point Likert scale (1 = totally; 10 = not at all) questionnaire composed of 4 items (1. During the pandemic, my life was controlled by some events on what I had… totally control-no control; 2. I had total control, no control over things that happened in my life during the pandemic; 3. How much did you feel in control of your life during the pandemic? 4. How much did you feel like an external spectator or principal actor in your life during the pandemic?) was used, and it was inspired by the study of Kay, Gaucher, Napier, Callan, and Laurin [28]. In this study, the questionnaire showed good internal reliability (Cronbach’s alpha = 0.80). The composite showed *M* = 24.52, *SD* = 6.90; inter-item correlations were moderate (0.34–0.68). Corrected item–total correlations (and α-if-deleted) indicated homogeneous items.

#### 2.2.4. Conspiracy Theories About COVID-19 and Vaccine

According to the main conspiracy theories present on the web during the pandemic, an ad hoc questionnaire was created to collect opinions on COVID-19-related issues and about COVID-19 vaccines. Participants rated their degree of agreement with COVID-19-related conspiracy theories through a 5-point Likert scale. Specifically, the three most frequent conspiracy theories about COVID-19 (“Covid was created in laboratory”; “COVID-19 was introduced by the governments to limit citizen personal freedom”; “COVID-19 is only a flu”) and about the vaccine (“the vaccine was introduced only to make Pharma richer”; “Physicians cannot tell the truth about vaccines because they could lose their work”; “The vaccine is more harmful than COVID-19”) were selected for the analysis. The questionnaire showed good internal reliability in this study (Cronbach’s alpha = 0.88) and inter-item correlations ranging 0.19–0.73, supporting unidimensional scoring.

#### 2.2.5. Adherence to Government Guidelines

Adherence to government guidelines on preventing and combating the spread of SARS-CoV-2 was measured through 8 items that assessed particular healthy behaviours through a 5-point Likert scale (1 = never; 5 = always) according to the frequency they implemented that given behaviour. Then, participants were to respond through 8 items according to their degree of agreement through a 5-point Likert scale (1 = totally disagree; 5 = totally agree). Higher scores indicate more adherence to government guidelines. This questionnaire showed acceptable internal reliability (Cronbach’s alpha = 0.75). The composite showed *M* = 21.32, *SD* = 4.67; inter-item correlations were positive overall (approx. 0.20–0.68). Corrected item–total correlations were computed and supported treating items as a coherent composite.

#### 2.2.6. Use of the Main Sources of Information: Frequency and Trust

An ad hoc questionnaire measured the types of information sources participants used and their trust in these sources. The sources listed included traditional ones, such as newspapers and TV newscasts, as well as web-based platforms like Facebook, Instagram, WhatsApp, and YouTube. The questionnaire was divided into two sections: The questionnaire was divided into two sections: in the first, the participants were asked to respond via a 5-point Likert Scale about the frequency they use those sources (from 1 = never to 5 = always) in the second, participants were asked if and how much they trust in those sources and from (1 = not at all to 5 = totally). This questionnaire provided four different scores: use of traditional sources of information, use of social media sources of information, trust in traditional sources of information, and trust in social media sources of information. This questionnaire showed acceptable internal reliability for both sections (Cronbach’s alpha = 0.70 and 0.73, respectively). Regarding trust in social media, the composite showed *M* = 7.22, *SD* = 2.88; inter-item correlations were 0.45–0.55. Item-total diagnostics supported the composite while regarding trust in traditional sources the composite showed *M* = 9.71, *SD* = 2.78; inter-item correlations were 0.43–0.60. Item-total diagnostics confirmed adequate homogeneity.

### 2.3. Statistical Analysis

Statistical analyses were performed using IBM SPSS Statistics for Windows, Version 25.0 (IBM Corp., Armonk, NY, USA). Gender was coded as a dummy variable: F = 0 and M = 1, as well as the educational status (primary = 1; secondary = 2; high school = 3; bachelor degree = 4; master degree = 5) and the belief in the different conspiracy theories (no = 0; yes = 1) Bivariate statistics such as Pearson r were used to explore the relationship between predictive variables, while univariate analysis of variance (ANOVA) and Chi-square test were conducted to explore scores differences in function of demographics. To address distributional assumptions, univariate normality of continuous variables was checked via skewness, excess kurtosis, and Shapiro–Wilk tests, recognizing that with *N* = 437 these tests are highly sensitive to minor departures. For linear models, normality was evaluated at the level of residuals, which showed approximate normality on standard diagnostics for the variables overall. Finally, serial multiple-mediator models were estimated with Hayes’ PROCESS macro (Model 6), using 5000 bias-corrected bootstrap resamples and 95% confidence intervals for indirect effects, an approach that is appropriate also when the sampling distribution of the product term is non-normal; therefore, parametric estimates were retained. For each conspiracy-belief predictor (COVID-19; vaccine), healthy behaviors (adherence) served as the outcome. We structured our mediation models to assess whether the influence of conspiracy beliefs on health behaviors was mediated through changes in perceived control and trust in different information sources. Specifically, we hypothesized that: Belief in conspiracy theories would negatively impact healthy behaviors. PROCESS macro for SPSS (Model 6) was chosen for its robustness in handling multiple mediators simultaneously, providing direct and indirect effects. This model allows estimation of serial multiple-mediation pathways, which is crucial for understanding the complex interplay among our variables of interest. Each model used a single conspiracy theory as its predictor.

## 3. Results

### 3.1. Descriptive Statistics and Demographics

Table 1 summarizes participants’ characteristics and endorsement rates. The sample was predominantly from South/Islands (79.6%), with 11.0% from the North and 9.4% from the Center of Italy; recruitment channels also yielded a student-heavy composition (52.4%), which is noted in the Limitations. Regarding conspiracy beliefs about COVID-19, 25.9% believe that “COVID-19 was created in a laboratory,” 7.4% that “COVID-19 is only the flu,” and 6.4% that “COVID-19 was introduced to limit citizens’ freedom.” For vaccine-related conspiracy beliefs, 16.5% believe that “the vaccine was created only to make Pharma richer,” 8.7% that “physicians cannot tell the truth about the vaccine,” and 4.6% that “the vaccine is more harmful than COVID-19.”

### 3.2. Correlational Analysis

The bivariate association between variables is reported in Table 2. Specifically, psychological well-being showed a positive and significant relationship with perceived control (*p* < 0.01) and trust in social media sources of information (*p* < 0.05). In contrast, no significant relationship with belief in all conspiracy theories were found (all *ps* > 0.05). As regards perceived control, positive and significant relationships were found with trust in social media source (*p* < 0.01) and negative and significant relationship was found with trust in traditional sources of information (*p* < 0.01). In contrast, no significant relationships were found with conspiracy theories except for one of them, but only marginally. As regards the trust in sources of information, traditional ones showed a significant and negative relationship with all conspiracy theories (all *ps* < 0.01), while trust in social media sources showed a significant and positive relationship with only conspiracy theories about vaccines. Finally, the adherence to government guidelines during the pandemic showed a positive and significant relationship with trust in traditional sources and a negative relationship with all conspiracy theories (all *ps* < 0.01).

### 3.3. Comparison Between Those Who Believe in Conspiracy Theories and Those Who Do Not

Table 3 presents data comparing the mean scores between individuals who believe in conspiracy theories and those who do not. Particularly, as regards well-being, no significant differences were found between those who believe in conspiracy theories and who do not (all *ps* > 0.05) as well as perceived control scores except for those who believe that COVID-19 was introduced to limit citizens’ personal freedom showing more control than those who not believe in this theory (*F* =3.89; *p* < 0.05). Regarding social media use, those who believe in conspiracy theories showed they used more social networks as a source of information than those who did not, while no differences were observed in the use of traditional sources of information. Differently, the trust in traditional information was significantly lower for those who believe in almost all conspiracy theories than those who do not (all *ps* < 0.01), and, at the same time, also the adherence to government guidance was lower in those who believe in the conspiracy theories (all *ps* < 0.01).

### 3.4. Mediation Analysis

**Figure 1:**
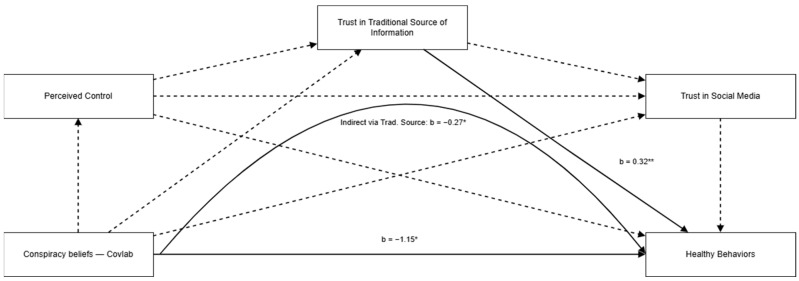
Mediation Model of the Relationship Between Conspiracy Theories and Healthy Behaviors-Model 1. Note. Above arrows: unstandardized b (main paths). Curved X → Y = indirect via Trust in Traditional. Solid = *p* < 0.05; dashed = n.s. * *p* < 0.05; ** *p* < 0.001.

Figure 1 illustrates the mediation model, in which conspiracy theories are negatively associated with healthy behaviors related to adherence to government guidance and perceived control, and trust in social media and traditional sources of information serves as a mediator of this relationship. For the sake of brevity, only model 1 is presented graphically, while all models are presented in the Supplemental Materials (Figure S1). Multiple models were run for each conspiracy theory, and the results are presented in Table 4. Specifically, regarding conspiracy theories about COVID-19, the models showed that belief in conspiracy theories negatively predicts healthy behaviors related to adherence to government guidance (*ps* < 0.001), except for Model 1 (COVID-19 was created in the laboratory). Moreover, in the other models (2–3), perceived control and trust in social media did not show significant associations, while only trust in traditional sources showed a significant association with the outcome variable. In the same way, regarding conspiracy theories about vaccines, Model 4 (Vaccine makes Pharma richer) did not show significant associations with the outcome variables, while the others (Models 5–6) showed that belief in these conspiracy theories negatively predicts healthy behaviors. All the significant models explained around 6–8% of the total variance. Finally, as regards the mediation effect, all significant models showed a significant indirect effect of conspiracy theories only through the trust in traditional sources of information and not through the paths of the other variables.

## 4. Discussion

The present study aimed to verify the relationship between beliefs in conspiracy theories and a lower perception of personal control, as well as how belief in conspiracy theories is related to lower adherence to health behaviors, such as vaccination campaigns. Thus, we also hypothesize that belief in conspiracy theories is most prevalent in those individuals who use and trust “non-traditional” sources of information, such as social media, and that perceived control and mistrust in traditional sources of information had a mediating role in the relationship between belief in conspiracy theories and health-related behaviors during the COVID-19 pandemic.

As regards socio-demographic variables, our findings show that individuals with a higher level of education (degree and master’s) are less likely to believe in conspiracy theories; this data are in line with other studies [20,29] and it is probably because higher levels of education ensure the development of analytical and critical thinking skills that provide the tools to recognize possible contradictions in conspiracy theories [30]. Regarding age, we found that as age increases, belief in conspiracy theories also increases. However, our findings are in contrast with some studies [5,21,31] that reported opposite results. Specifically, a study by Galliford & Fuhrman [31] suggests that because younger people are more likely to be exposed to non-traditional information sources, they are also more likely to be exposed to conspiracy theories often spread through social media. For those who regard our study, a possible explanation is that, in our sample, most participants were college students or postgraduates, so they were both young and highly educated.

Regarding the different conspiracy theories about COVID-19, most of the sample reported believing that it was created in a laboratory, while the rest of the sample believed, respectively, that it was only the flu and that COVID-19 was introduced to limit citizens’ personal freedom. Regarding vaccine conspiracy theories, most of the sample reported believing that the vaccine was created solely to enrich pharmaceutical companies.

In comparing the mean scores between those who believe in conspiracy theories and those who do not, we found some differences based on the type of theories people believe in. Specifically, only those who believe that COVID-19 was introduced to limit citizens’ personal freedom showed more control than those who do not believe in this theory, while this difference was not found among the other theories. On the contrary, believing in conspiracy theories in general seems to be related to being less likely to engage in healthy behaviours and being more likely to use social media. People believing in conspiracy theories also reported low trust in traditional sources of information. These findings are consistent with previous studies [6,21] that highlight the role of the sources of information in the spread of conspiracy theories, as well as related risky behaviours.

Regarding correlation analysis, our findings showed a positive and significant relationship between psychological well-being and high levels of perceived control, but no significant relationship with belief in all conspiracy theories considered. While it is easily intuited that greater perceived control over one’s life correlates with greater well-being, it is unexpected that there is no negative correlation between well-being and belief in conspiracy theories, as we had also hypothesized based on the results of previous studies [14,18]. We put forward two possible explanations: first, since this is a cross-sectional study, it is possible that at the time of data collection there had not yet been an effect on the size of well-being in those who believed in conspiracy theories; second, ours is a nonclinical sample and most of the participants scored medium to high in well-being, so other variables are probably involved. Regarding perceived control, we found a positive relationship with trust in social media sources and a negative relationship with trust in traditional sources of information, while no significant relationship was found with conspiracy theories overall. The lack of a relationship between belief in conspiracy theories and perceived control contrasts with our first hypothesis, which predicted that belief in conspiracy theories was related to a lower perceived control.

However, data in the literature about perception control and beliefs in conspiracy theories are complex. Van Proojen and Acker [32] suggest that people’s ability to exert control over the social environment, especially in those situations that are characterized by feelings of threat, is linked with their desire to make sense of it, which is a central ingredient of belief in conspiracy theories. Moreover, Social identity dynamics [33] can contribute to the polarization of trust, as alignment with group narratives reinforces a sense of belonging and self-consistency. In addition to this, adherence to a conspiracy theory can also reduce the dissonance arising from feelings of powerlessness, mistrust, or exclusion. Believing in a conspiracy offers an orderly and coherent narrative of the world, in which ‘we’ (the lucid, the informed) have understood what ‘they’ (the elites, the institutions) are hiding. This process reduces the dissonance between the need for control and the experience of uncertainty or loss of trust in institutions [34]. A plausible interpretation of our findings is that, at the time of data collecting, the beliefs in conspiracy theories had already acted as a compensatory mechanism for lack of control’s feelings; but due to the nature of our research design, which did not involve the comparison of the levels of perceived control with a neutral base-rate condition, we cannot state this as evidence. In addition, the fact that our study is based primarily on a sample of college students does not allow us to generalize the results to a broader population.

In line with previous research [30,35], our findings concerning the positive and negative associations between perceived control and trust in information sources support the view that personal beliefs are shaped by the tendency to align with pre-existing attitudes and convictions. This interpretation is consistent with the motivated reasoning framework [36], which suggests that individuals’ evaluations of information are guided by prior beliefs and emotional investments rather than accuracy of facts. Within this perspective, social media may act as powerful amplifiers of individuals’ core beliefs, as well as of their distrust toward traditional and institutional sources.

Moreover, the algorithm behind social media creates a kind of cyber bubble in which information is shared within a homogeneous circle of people who are bound by the same cultural background, interests, and beliefs [35]. In this way, it may be possible to assume that through the use of social media, we select the news that does not hurt our mental well-being and that reinforces our perception of control [37]. On the other hand, the widespread conspiracy theories on social media fuel mistrust of traditional information sources that are perceived as part of the conspiracy. Indeed, we found a significant and negative relationship between the use of traditional sources of information and all conspiracy theories. According to Jennings and colleagues [30], misinformation spread in a context of lack of trust in government, politics, and elites, with important consequences on adherence to health-related behaviors. In line with the existing literature [38,39], our results showed that belief in conspiracy theories causes individuals to engage in potentially risky behaviors such as not adhering to vaccination campaigns and, in addition, not adhering to government guidelines regarding countering the COVID-19 pandemic. Going more in-depth into these mechanisms, if we looked at the mediation effect, all significant models showed that conspiracy theories negatively predict healthy behaviors primarily through trust in traditional sources of information, rather than through perceived control or trust in social media. This suggests that trust in traditional sources and institutions is the most significant predictor of adherence to health-related behaviors. This result can be interpreted through the lens of compensatory control theory [28] according to which when individuals experience a decrease in personal control, they may compensate by strengthening their trust in external systems that provide structure and predictability. Thus, rather than acting as a mediator per se, perceived control may indirectly influence attitudes through reliance on conventional or institutional sources that restore a sense of order.

Moreover, these results align with other studies that underline how trust in the source of information suggests that individuals who believe in conspiracy theories are more likely to distrust official or mainstream sources. This mistrust likely stems from a perception that these sources are part of the conspiracy. As a result, individuals who hold such beliefs may disregard health guidelines and recommendations issued by these sources, perceiving them as unreliable and deceptive, also in countries where people are not trained to check their scientific truthfulness [40]. This finding aligns with previous research indicating that mistrust in government and official institutions is a critical factor in spreading conspiracy theories and related health behaviors [30]. Although we hypothesized that trust in social media would play a significant mediating role, our results did not support this. One possible explanation is that while social media serves as a platform for spreading conspiracy theories, it does not directly influence individuals’ health behaviors as strongly as their underlying mistrust in traditional sources does. Social media may amplify and perpetuate conspiracy beliefs, but the decision to reject health guidelines seems to be more closely tied to a lack of trust in the traditional sources that promote these guidelines.

Moreover, contrary to our expectations, perceived control did not significantly mediate the relationship between conspiracy beliefs and health behaviors. This may be because the sense of control conferred by conspiracy theories operates at a psychological level, providing a framework for understanding the world rather than directly impacting specific health behaviors. Belief in conspiracy theories might act as a coping mechanism to deal with uncertainty and fear, without necessarily translating into tangible actions like rejecting vaccines or health guidelines. Trust in traditional sources of information regulates the credibility of health recommendations, and in case of low trust in this information, individuals could seek alternative explanations that align with their beliefs [40]. This dynamic underscores the importance of trust-building in public health communications, particularly in countering the spread of misinformation. Finally, cultural and contextual factors may also play a role in these mediation effects. In contexts where trust in government and institutions is already low, such as Italy, conspiracy theories find fertile ground, and this pre-existing mistrust may be more influential than any sense of control provided by these theories. Regarding this, it is reasonable to assume that the recurring political crises, which have led to the instability of recent governments and the consequent fluctuations in institutional credibility, have contributed to creating a certain ambivalence toward government institutions. This persistent mistrust may make Italian citizens more susceptible to alternative explanations and conspiracy theories, particularly in times of uncertainty or crisis. In this sense, placing our findings within this socio-cultural framework reinforces the ecological validity of the study and highlights the role of cultural factors in shaping both trust and susceptibility to disinformation.

It should also be noted that the present findings primarily reflect a young and highly educated sample, which suggests that the behavioral patterns observed may not fully generalize to the broader population. Therefore, interpretations regarding public health implications should be made with caution.

It should be emphasized that, due to the cross-sectional nature of the study, the associations observed cannot be interpreted as causal. The proposed mechanisms linking conspiracy beliefs, trust in information sources, and adherence to health recommendations should therefore be considered theoretical hypotheses that warrant confirmation through longitudinal or experimental research designs.

These findings highlight the need for communication strategies based on psychological knowledge during large-scale crises, in which the trustworthiness and credibility of messages are mediated by new and non-traditional sources of information.

### Limitations and Future Directions

This study suffers from some limitations: first of all, the cross-sectional research design does not allow for a comparison of the levels of perceived control and well-being during the pandemic with a neutral base-rate condition, and to grasp the effect of the changes in the variables over time. Second, our initial sample is mostly represented by college students, especially psychology students, so it is possible that the broader sample was influenced, as participants were likely to recruit individuals, via snowball sampling, from similar demographics or social circles. Moreover, the sample shows a marked South/Islands prevalence (79.6%) and this macro-regional imbalance may also mirror temporal heterogeneity in the spread of COVID-19 and in local containment measures, potentially shaping perceived threat and exposure to public messaging; future studies should address this by using stratified sampling or post-stratification and, where possible, including time/region controls. This limits the generalizability of the findings to the broader population. For these reasons, the behavioral implications discussed should therefore be considered preliminary and specific to similar demographic groups. In addition, the use of self-reports only may be associated with common method bias, such as social desirability bias. Finally, some other variables (personal and/or situational) that should influence the results could be excluded from the analysis and not controlled (for example, personality traits).

## 5. Conclusions

Despite the growing body of studies that have investigated the psychological factors that are associated with belief in conspiracy theories and the role they play in inhibiting health-related behaviors in the specific context of the COVID-19 pandemic, to our knowledge no study has yet considered the mediating role of trust in sources of information in the already proven negative relationship between belief in conspiracy theories and health-related behaviors, particularly concerning the vaccination campaign’s adherence. We strongly believe that, beyond individual variables, what most negatively affected the response to government public health guidelines was mistrust in traditional sources of information and, by extension, in institutions and government.

Misinformation spreads more easily in contexts of low institutional trust. Thus, transparent and psychologically informed communication is essential during emergencies. When communication is perceived as unclear or overwhelming, people may seek alternative explanations to regain a sense of control.

The spread of conspiracy theories through social media is facilitated by the fact that to post content on a social platform (such as YouTube, TikTok, Instagram, Facebook, or Twitter), it is not necessary to provide proof of the veracity of the sources. It should also be noted that social media offers a more interactive and participatory way to access information, fostering a sense of belonging and agency. This dynamic may help individuals compensate for feelings of powerlessness sometimes triggered by traditional media outlets, which may rely on alarmist communication strategies to capture attention.

In practical terms, our findings highlight the need to strengthen public trust and resistance to misinformation. Evidence-based interventions such as media-literacy programs, which help individuals critically evaluate online content, and inoculation-based strategies, which expose individuals to weakened forms of misinformation to build psychological resistance, may be effective tools in preventing the spread of conspiracy beliefs and improving adherence to health recommendations. Moreover, crisis-communication frameworks that emphasize transparency, emotional validation, and consistent messaging have been shown to increase institutional trust and compliance with public health guidance. Integrating these approaches into public health strategies may help support resilience against future infodemics and improve public cooperation during health crises.

As this study is cross-sectional, the patterns reported here represent associations rather than causal pathways; future longitudinal and experimental research is needed to validate the hypothesized mechanisms.

These findings suggest that government communication strategies should integrate social media channels and be informed by psychological insights into trust and perception, in order to mitigate the spread of conspiracy theories and their potential public health consequences.

## Figures and Tables

**Table 1:** Demographics and Frequencies of Conspiracy Theories in the Sample.

Demographic Information	N (%)
Gender	
M	140 (32.0)
F	296 (67.7)
Age	31.4 (13.3)
Region	
North	48 (11.0)
Centre	41 (9.4)
South/Islands	348 (79.6)
Marital status	
Single	305 (69.8)
Married	117 (26.8)
Divorced	13 (3.0)
Widowed	2 (0.5)
Education	
Primary	0 (0.0)
Secondary	30 (6.9)
High School	232 (53.1)
Bachelor	109 (24.9)
Master	66 (15.1)
COVID-19 was created in laboratory	
No	324 (74.1)
Yes	113 (25.9)
COVID-19 to limit personal freedom	
No	409 (93.6)
Yes	28 (6.4)
COVID-19 is only a flu	
No	405 (92.7)
Yes	32 (7.3)
The vaccine was created to make Pharma richer	
No	365 (83.5)
Yes	72 (16.5)
Physicians cannot tell the truth about the vaccine	
No	399 (91.3)
Yes	38 (8.7)
The vaccine is more harmful than COVID-19	
No	417 (95.4)
Yes	20 (4.6)

Note. Frequencies of demographic information and different conspiracy theories distribution in the sample n (%).

**Table 2:** Correlational Analysis Between Variables.

Variables	1	2	3	4	5	6	7	8	9	10
1. Sex										
2. Age	0.06	1								
3. Education	0.06	0.06	1							
4. Well-being	0.24 **	0.21 **	0.02	1						
5. Perceived control	0.23 **	0.26 *	0.05	0.43 **	1					
6. Adherence to Gov. Guidelines	0.18	0.11 *	0.09	0.06	0.02	1				
7. Use of traditional sources	0.09	0.12	0.03	0.01	0.00	0.16	1			
8. Trust in trad. sources	0.02	0.17	0.11 *	0.06	0.11 *	0.19 **	0.24 **	1		
9. Trust Soc. Media	0.07	0.07	0.02	0.10	0.13	0.06	0.10 *	0.17 **	1	
10. Social Media sources	0.01	0.04	0.00	0.08	0.11 *	0.03	0.02	0.15 **	0.57 **	1
11. COVID-19 lab	0.04	0.16 **	0.12 *	0.00	0.05	−0.14 **	0.01	0.13 **	0.09	0.11 *
12. Limit. Pers. Freed.	0.02	0.18 **	0.06	0.05	0.09 *	−0.24 **	0.06	0.21 **	0.09	0.03
13. COVID-19 is a flu	0.04	0.02	0.03	0.04	0.04	−0.20 **	0.00	0.07	0.03	0.01
14. Vaccine threat	0.05	0.16 **	0.08	0.02	0.05	−0.18 **	−0.07	0.19 **	−0.21 **	0.18 **
15. Vaccine makes Pharmacies richer	−0.01	0.18 **	−0.15 **	−0.00	0.08	−0.21 **	0.05	−0.14 **	0.07	0.07

Note. For the sake of clarity only correlation between variables and Conspiracy Theories are reported. Significance at * *p* < 0.05 and ** *p* < 0.01.

**Table 3:** Mean(SD) Comparison Between Groups of who Believe in Conspiracy Theories and Who do not.

Measure	COVID-19 Created in a Lab			Personal Freedom			COVID-19 Is a Flu			Vaccine Makes Pharma Richer			Physicians Cannot Tell the Truth			Vaccine is More Harmful Than COVID-19		
	**No**	**Yes**	**F**	**No**	**Yes**	**F**	**No**	**Yes**	**F**	**No**	**Yes**	**F**	**No**	**Yes**	**F**	**No**	**Yes**	**F**
Well-being	70.00 (±14.50)	70.19 (±15.45)	0.01	69.84 (±14.68)	73.07 (±15.25)	1.25	69.86 (±14.57)	72.43 (±16.56)	0.90	69.98 (±14.79)	71.93 (±12.84)	0.25	70.06 (±14.66)	70.00 (±15.17)	0.01	70.28 (±14.59)	67.60 (±16.09)	1.15
Perceived Control	24.31 (±6.73)	25.09 (±7.37)	1.07	24.34 (±6.84)	27.00 (±7.46)	3.89 *	24.43 (±6.80)	25.56 (±8.13)	0.79	24.44 (±6.88)	26.66 (±7.37)	1.50	24.26 (±6.93)	25.82 (±6.64)	3.01	24.44 (±6.95)	25.28 (±6.42)	0.52
Social media (use)	9.68 (±3.85)	10.67 (±4.00)	5.38 *	9.90 (±3.87)	10.39 (±4.54)	0.39	9.92 (±3.89)	10.15 (±4.24)	0.10	9.84 (±3.86)	12.73 (±4.33)	8.04 *	9.80 (±3.89)	10.62 (±3.98)	2.64	9.80 (±3.91)	11.31 (±3.61)	5.19 *
Traditional sources (use)	7.82 (±2.64)	7.92 (±3.12)	0.10	7.85 (±2.68)	7.78 (±3.86)	0.01	7.85 (±2.74)	7.78 (±3.09)	0.02	7.88 (±2.72)	6.80 (±3.78)	2.22	7.78 (±2.63)	8.18 (±3.37)	1.23	7.78 (±2.68)	8.47 (±3.53)	2.11
Trust in Social media	7.07 (±2.73)	7.66 (±3.25)	3.57	7.15 (±2.78)	8.25 (±4.30)	3.84	7.19 (±2.87)	7.62 (±2.95)	0.66	7.12 (±2.70)	10.00 (±5.43)	14.90 **	7.12 (±2.77)	7.70 (±3.31)	2.44	7.11 (±2.75)	8.42 (±3.76)	7.30 *
Trust in Trad. Sources	9.94 (±2.60)	9.05 (±3.16)	8.61 **	9.86 (±2.69)	7.42 (±3.09)	20.99 **	9.76 (2.76)	8.96 (2.97)	2.45	9.82 (±2.69)	6.53 (±3.35)	21.14 **	9.88 (±2.64)	8.81 (±3.30)	8.96 *	9.87 (±2.66)	7.92 (±3.33)	17.82 **
Adherence to Gov. Guid.	21.71 (±4.33)	20.19 (±5.38)	9.05 **	21.61 (±4.39)	17.00 (±6.30)	27.17 **	21.58 (±4.36)	18.00 (±6.75)	18.16 **	21.48 (±4.53)	16.80 (±6.16)	15.03 **	21.75 (±4.39)	19.11 (±5.39)	20.28 **	21.56 (±4.47)	18.78 (±5.86)	12.57 **

Note. Significance at * *p* < 0.05 and ** *p* < 0.01.

**Table 4:** Mediation models with Healthy Behaviors as Dependent Variable.

Model	Predictors	R^2^	F	B	SE	95%CI
Model 1	Direct effects	0.06	6.01 *			
	CovLab → HealthyB			−1.15 *	0.50	[−2.14; −0.17]
	TradTrust → HealthyB			0.32 ***	0.08	[0.16; 0.48]
	Indirect effect					
	CovLab → TradTrust → HealthyB			−0.27 *	0.11	[−0.50; −0.04]
Model 2	Direct effects	0.08	14.56 **			
	LimitPersFreed → HealthyB			−3.86 ***	0.90	[−5.63; −2.09]
	TradTrust → HealthyB			0.27 ***	0.08	[0.11; 0.43]
	Indirect effects					
	LimitPersFreed → TradTrust → HealthyB			−0.63 **	0.23	[−1.10; −0.16]
Model 3	Direct effects	0.08	12.89 **			
	CovFlu → HealthyB			−3.27 ***	0.82	[−4.89; −1.65]
	TradTrust → HealthyB			0.32 ***	0.07	[0.17; 0.48]
Model 4	Direct effects	0.07	10.48 **			
	VaxPharmaRich → HealthyB			−2.26 ***	0.58	[−3.41; −1.11]
	TradTrust → HealthyB			0.30 ***	0.08	[0.14; 0.46]
	Indirect effects					
	VaxPharmaRich → TradTrust → HealthyB			−0.30 *	0.13	[−0.56; −0.04]
Model 5	Direct effects	0.06	13.32 **			
	VaxHarmful → HealthyB			−2.64 *	1.1	[−4.80; −0.49]
	TradTrust → HealthyB			0.30 ***	0.08	[0.13; 0.46]
	Indirect effects					
	VaxHarmful → TradTrust → HealthyB			−0.08 **	0.28	[−1.36; −0.23]
Model 6	Direct effects	0.06	11.04 **			
	PhysiciansCan’t TellTruth → HealthyB			−2.01 *	0.79	[−3.57; −0.45]
	TradTrust → HealthyB			0.30 **	0.08	[0.14; 0.46]
	Indirect effects					
	PhysiciansCan’t TellTruth → TradTrust → HealthyB			−0.58 **	0.21	[−0.99; −0.17]

Note: Multiple Mediation models with every conspiracy theory as predictors of healthy behaviors and Perceived control, trust in traditional sources of information and social media as mediatiors * *p* < 0.05; ** *p* < 0.01; *** *p* < 0.001 Note. HealthyB, healthy behavior; CovLab, COVID-19 was created in a laboratory; TradTrust, trust in traditional sources; LimitPersFreed, COVID-19 limits personal freedom; CovFlu, COVID-19 is a flu; VaxPharmaRich, vaccine make Pharma richer; VaxHarmful, vaccine is more harmful than COVID-19; PhysiciansCan’t TellTruth, physicians cannot tell the truth. For the sake of clarity, only significant paths between predictors and the outcome variable are reported in the table.

## Data Availability

The raw data supporting the conclusions of this article will be made available by the authors on request.

## Supplementary Materials

The following supporting information can be downloaded at: https://www.mdpi.com/article/10.3390/healthcare13222915/s1, Figure S1. Mediation Model of the Relationship Between Conspiracy Theories and Healthy Behaviors-All Models.

## References

[1] Salameh G., Marais D., Khoury R. (2023). Impact of COVID-19 Pandemic on Mental Health among the Population in Jordan. Int. J. Environ. Res. Public Health.

[2] van Prooijen J.W., Douglas K.M. (2017). Conspiracy theories as part of history: The role of societal crisis situations. Mem. Stud..

[3] Loomba S., de Figueiredo A., Piatek S.J., de Graaf K., Larson H.J. (2021). Author Correction: Measuring the impact of COVID-19 vaccine misinformation on vaccination intent in the UK and USA. Nat. Hum. Behav..

[4] World Health Organization (2022). Coronavirus Disease (COVID-19) Advice for the Public: Mythbusters.

[5] Freeman D., Lambe S., Yu L.-M., Freeman J., Chadwick A., Vaccari C., Waite F., Rosebrock L., Petit A., Vanderslott S. (2023). Injection fears and COVID-19 vaccine hesitancy. Psychol. Med..

[6] Earnshaw V.A., Eaton L.A., Kalichman S.C., Brousseau N.M., Hill E.C., Fox A.B. (2020). COVID-19 conspiracy beliefs, health behaviors, and policy support. Transl. Behav. Med..

[7] De Coninck D., Frissen T., Matthijs K., d’Haenens L., Lits G., Champagne-Poirier O., Carignan M.-E., David M.D., Pignard-Cheynel N., Salerno S. (2021). Beliefs in Conspiracy Theories and Misinformation About COVID-19: Comparative Perspectives on the Role of Anxiety, Depression and Exposure to and Trust in Information Sources. Front. Psychol..

[8] Islam M.S., Sarkar T., Khan S.H., Mostofa Kamal A.H., Hasan S.M.M., Kabir A., Yeasmin D., Islam M.A., Chowdhury K.I.A., Anwar K.S. (2020). COVID-19-Related Infodemic and Its Impact on Public Health: A Global Social Media Analysis. Am. J. Trop. Med. Hyg..

[9] Wake A.D. (2021). The Willingness to Receive COVID-19 Vaccine and Its Associated Factors: “Vaccination Refusal Could Prolong the War of This Pandemic”—A Systematic Review. Risk Manag. Healthc. Policy.

[10] Neumann-Böhme S., Varghese N.E., Sabat I., Barros P.P., Brouwer W., Van Exel J., Schreyögg J., Stargardt T. (2020). Once we have it, will we use it? A European survey on willingness to be vaccinated against COVID-19. Eur. J. Health Econ..

[11] Fedele F., Aria M., Esposito V., Micillo M., Cecere G., Spano M., De Marco G. (2021). COVID-19 vaccine hesitancy: A survey in a population highly compliant to common vaccinations. Hum. Vaccin. Immunother..

[12] Larson H.J., De Figueiredo A., Xiahong Z., Schulz W.S., Verger P., Johnston I.G., Cook A.R., Jones N.S. (2016). The State of Vaccine Confidence 2016: Global Insights Through a 67-Country Survey. EBioMedicine..

[13] Al-Amer R., Maneze D., Everett B., Montayre J., Villarosa A.R., Dwekat E., Salamonson Y. (2022). COVID-19 vaccination intention in the first year of the pandemic: A systematic review. J. Clin. Nurs..

[14] van Prooijen J.W., Etienne T.W., Kutiyski Y., Krouwel A.P.M. (2023). Conspiracy beliefs prospectively predict health behavior and well-being during a pandemic. Psychol. Med..

[15] Hornsey M.J., Bierwiaczonek K., Sassenberg K., Douglas K.M. (2023). Individual, intergroup and nation-level influences on belief in conspiracy theories. Nat. Rev. Psychol..

[16] Alsuhibani A., Shevlin M., Freeman D., Sheaves B., Bentall R.P. (2022). Why conspiracy theorists are not always paranoid: Conspiracy theories and paranoia form separate factors with distinct psychological predictors. PLoS ONE.

[17] Whitson J.A., Galinsky A.D. (2008). Lacking control increases illusory pattern perception. Science.

[18] Chen X., Zhang S.X., Jahanshahi A.A., Alvarez-Risco A., Dai H., Li J., Ibarra V.G. (2020). Belief in a COVID-19 Conspiracy Theory as a Predictor of Mental Health and Well-Being of Health Care Workers in Ecuador: Cross-Sectional Survey Study. JMIR Public Health Surveill..

[19] Leibovitz T., Shamblaw A.L., Rumas R., Best M.W. (2021). COVID-19 conspiracy beliefs: Relations with anxiety, quality of life, and schemas. Pers. Individ. Dif..

[20] Georgiou N., Delfabbro P., Balzan R. (2020). COVID-19-related conspiracy beliefs and their relationship with perceived stress and pre-existing conspiracy beliefs. Pers. Individ. Dif..

[21] Allington D., Dhavan N. (2020). The Relationship Between Conspiracy Beliefs and Compliance with Public Health Guidance with Regard to COVID-19.

[22] Goreis A., Voracek M. (2019). A Systematic Review and Meta-Analysis of Psychological Research on Conspiracy Beliefs: Field Characteristics, Measurement Instruments, and Associations with Personality Traits. Front. Psychol..

[23] Tsoy D., Tirasawasdichai T., Kurpayanidi K. (2021). Role of social media in shaping public risk perception during COVID-19 pandemic: A theoretical review. Int. J. Manag. Sci. Bus. Res..

[24] Nazli S.B., Yigman F., Sevindik M., Deniz Ozturan D. (2022). Psychological factors affecting COVID-19 vaccine hesitancy. Ir. J. Med. Sci..

[25] Zimmerman R.K., Wolfe R.M., Fox D.E., Fox J.R., Nowalk M.P., Troy J.A., Sharp L.K. (2005). Vaccine criticism on the World Wide Web. J. Med. Internet Res..

[26] Shahsavari S., Holur P., Wang T., Tangherlini T.R., Roychowdhury V. (2020). Conspiracy in the time of corona: Automatic detection of emerging COVID-19 conspiracy theories in social media and the news. J. Comput. Soc. Sci..

[27] Grossi E., Groth N., Mosconi P., Cerutti R., Pace F., Compare A., Apolone G. (2006). Development and validation of the short version of the Psychological General Well-Being Index (PGWB-S). Health Qual. Life Outcomes.

[28] Kay A.C., Gaucher D., Napier J.L., Callan M.J., Laurin K. (2008). God and the government: Testing a compensatory control mechanism for the support of external systems. J. Personal. Soc. Psychol..

[29] Constantinou M., Gloster A.T., Karekla M. (2021). I won’t comply because it is a hoax: Conspiracy beliefs, lockdown compliance, and the importance of psychological flexibility. J. Context. Behav. Sci..

[30] Jennings W., Stoker G., Bunting H., Valgarðsson V.O., Gaskell J., Devine D., McKay L., Mills M.C. (2021). Lack of Trust, Conspiracy Beliefs, and Social Media Use Predict COVID-19 Vaccine Hesitancy. Vaccines.

[31] Galliford N., Furnham A. (2017). Individual difference factors and beliefs in medical and political conspiracy theories. Scand. J. Psychol..

[32] Van Prooijen J., Acker M. (2015). The influence of control on belief in conspiracy theories: Conceptual and applied extensions. Appl. Cogn. Psychol..

[33] Tajfel H., Turner J.C., Austin W.G., Worchel S. (1979). An integrative theory of intergroup conflict. The Social Psychology of Intergroup Relations.

[34] Festinger L. (1957). A Theory of Cognitive Dissonance.

[35] Park Y., Chung J., Kim J. (2022). Social media, misinformation, and cultivation of informational mistrust: Cultivating Covid-19 mistrust. Journalism.

[36] Kunda Z. (1990). The case for motivated reasoning. Psychol. Bull..

[37] Jang S., Kim J. (2018). Third person effects of fake news: Fake news regulation and media literacy interventions. Comput. Hum. Behav..

[38] Jolley D., Douglas K.M. (2014). The effects of anti-vaccine conspiracy theories on vaccination intentions. PLoS ONE.

[39] Yang Z., Luo X., Jia H. (2021). Is It All a Conspiracy? Conspiracy Theories and People’s Attitude to COVID-19 Vaccination. Vaccines.

[40] Mubarak N., Rana F., Khan A. (2022). The conundrum of new coronavirus variants and poor uptake of booster dose: Building a narrative against vaccine hesitancy in Pakistan. J. Univ. Med. Dent. Coll..

