# Supplementary material for: Exploring the Relationship Between Conspiracy Theory Beliefs and Adherence to Government Guidelines During the COVID-19 Pandemic: The Role of Perceived Control and Trust in Social Media and Traditional Sources of Information
Source: Healthcare (Basel). 2025 Nov 14;13(22):2915. doi: 10.3390/healthcare13222915 (PMC12652441; doi:10.3390/healthcare13222915)
Supplement: Supplementary file 1 [file healthcare-13-02915-s001.zip › healthcare-3951818-supplementary.pdf]

Figure S.1 Mediation Model of the Relationship Between Conspiracy Theories and Healthy Behaviors- All Models

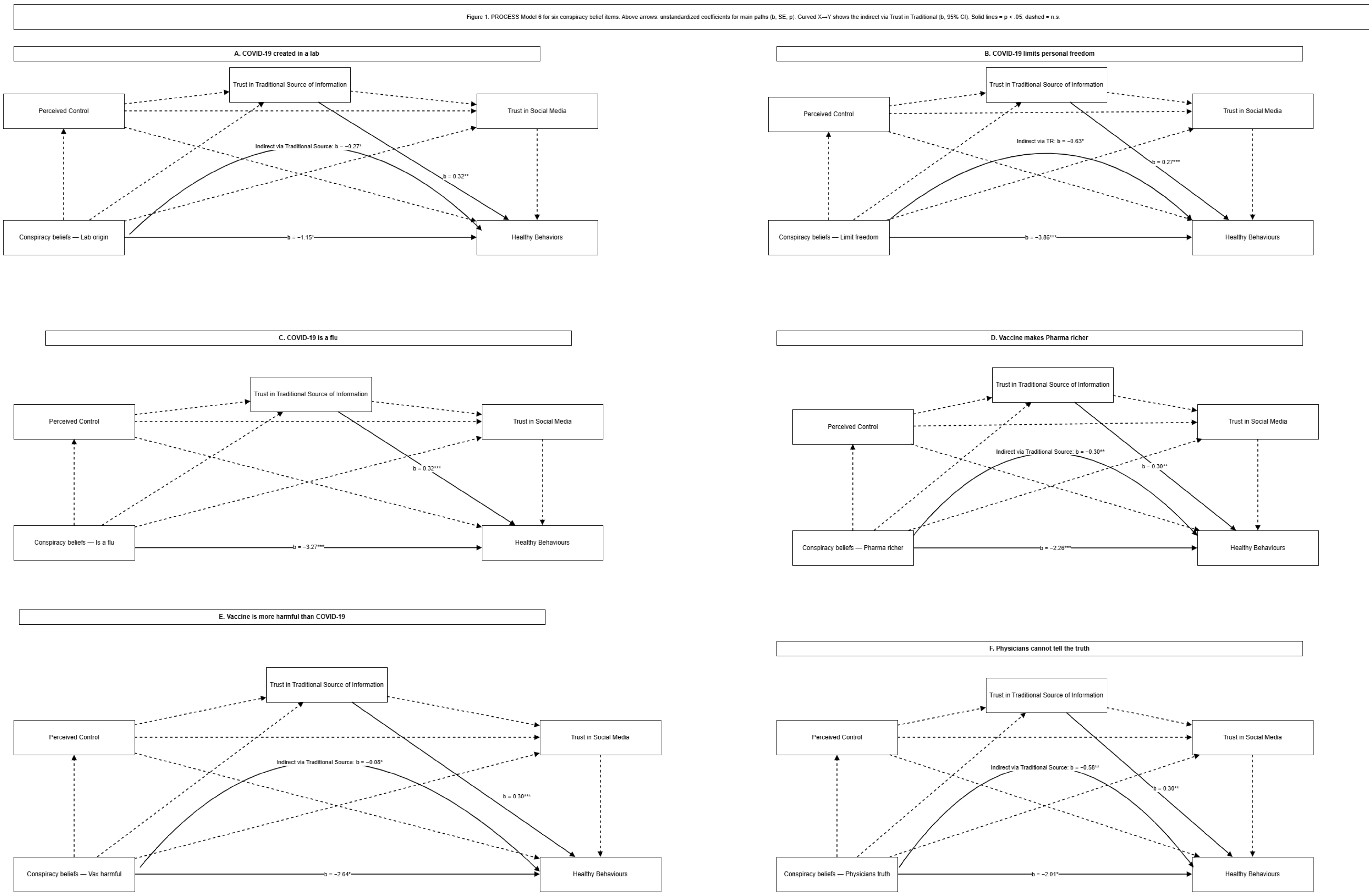

Note. Above arrows: unstandardized b (main paths). Curved X→Y = indirect via Trust in Traditional Solid = p < .05; dashed = n.s.
